# The Effect of Pedal Pump Lymphatic Technique Versus Passive Recovery Following Maximal Exercise: A Randomized Cross-Over Trial

**DOI:** 10.1186/s40798-021-00402-x

**Published:** 2022-01-15

**Authors:** Joanne DiFrancisco-Donoghue, Thomas Chan, Alexandra S. Jensen, James E. B. Docherty, Rebecca Grohman, Sheldon C. Yao

**Affiliations:** 1 Department of Osteopathic Manipulative Medicine, New York Institute of Technology College of Osteopathic Medicine (NYIT-COM), Northern Blvd, Old Westbury, NY 11568 USA; 2 Department of Clinical Specialties, NYIT-COM, Old Westbury, NY USA; 3 NYIT-COM Osteopathic Medical Student, Old Westbury, NY USA; 4 NYIT-COM Center for Sports Medicine, Old Westbury, NY USA

**Keywords:** OMM, Pedal pump, Recovery, Oxygen uptake

## Abstract

**Context:**

Muscle damage and delayed onset muscle soreness (DOMS) can occur following intense exercise. Various modalities have been studied to improve blood lactate accumulation, which is a primary reason for DOMS. It has been well established that active recovery facilitates blood lactate removal more rapidly that passive recovery due to the pumping action of the muscle. The pedal pump is a manual lymphatic technique used in osteopathic manipulative medicine to increase lymphatic drainage throughout the body. Pedal pump has been shown to increase lymphatic flow and improve immunity. This may improve circulation and improve clearance of metabolites post-exercise.

**Objective:**

This study compared the use of pedal pump lymphatic technique to passive supine recovery following maximal exercise.

**Methods:**

17 subjects (male *n* = 10, age 23 ± 3.01; female *n* = 7, age 24 ± 1.8), performed a maximal volume O_2_ test (VO_2_ max) using a Bruce protocol, followed by a recovery protocol using either pedal pump technique or supine passive rest for 10 min, followed by sitting for 10 min. Outcome measures included blood lactate concentration (BL), heart rate (HR), systolic blood pressure (SBP) and VO_2_. Subjects returned on another day to repeat the VO_2_ max test to perform the other recovery protocol. All outcomes were measured at rest, within 1- minute post-peak exercise, and at minutes 4, 7, 10 and 20 of the recovery protocols. A 2 × 6 repeated measures ANOVA was used to compare outcome measures (*p* ≤ 0.05).

**Results:**

No significant differences were found in VO_2_, HR, or SBP between any of the recovery protocols. There was no significant difference in BL concentrations for recovery at minutes 4, 7, or 10 (*p* > 0.05). However, the pedal pump recovery displayed significantly lower BL concentrations at minute 20 of recovery (*p* = 0.04).

**Conclusion:**

The pedal pump significantly decreased blood lactate concentrations following intense exercise at recovery minute 20. The use of manual lymphatic techniques in exercise recovery should be investigated further.

## Key Points

- The pedal pump lymphatic technique decreased blood lactate concentrations after 20 min of recovery following high intensity exercise compared to a passive recovery.
- Performing the pedal pump at a high frequency of propulsions per minute and low force was more effective than passive recovery.
- Manual lymphatic techniques may offer a more practical treatment option than other alternative methods currently being used in sports medicine.

## Introduction

Skeletal muscle energy consumption increases dramatically when going from rest to intense exercise [1, 2]. This high intensity exercise exceeds aerobic capacity and the ATP utilized for energy is derived from anaerobic metabolism. The anaerobic breakdown of glycogen leads to an accumulation of lactic acid. The byproduct of lactic acid is an excess of H^−^ and lactate. The H^−^ creates acidosis in the muscle and is a primary cause for reducing action potential and muscle fatigue [2]. Accumulation of lactic acid can be in part the cause of both acute and delayed muscle pain and attenuated muscle strength in the recovery period [1, 2]. This condition is called Delayed Onset Muscle Soreness (DOMS) [3]. In athletes who participate in intense exercise, DOMS can be frequent and managed in a variety of ways. However, exercise intensity is relative to each individual, therefore DOMS can present itself in untrained populations or patients in physical therapy as well. Post exercise pain and decreases in muscle strength during the recovery period can be debilitating, affect performance and negatively impact athletes’ and patients’ lives and discourage future exercise.

Decreasing blood lactate levels following exercise is a primary marker of recovery [3]. Active recovery methods (e.g. walking, biking) are more effective than passive recovery when trying to reduce blood lactate levels. This is in part due to the muscle pump mechanism that is promoted with active recovery [4, 5]. Venous-muscular pumps in the lower limb begin with the foot pump, followed by the calf pump that can be split into two leg pump locations. One is in the veins of the soleus muscle and the other is the popliteal pump ending in the popliteal vein. During rhythmic exercise, these lower leg pumping mechanism is a result of the muscle contracting and squeezing the blood out of the venous system back to the heart and prevent blood flowing back after each contraction decreasing venous pressure [6, 7]. Hence, the cardiovascular system and lymphatic system operate together to increase oxygen to all cells and remove waste products, including blood lactate accumulation following intense activity. This is the theory. Many strategies are used currently to enhance recovery post exercise with very little scientific evidence to demonstrate effectiveness.

Manual lymphatic drainage techniques (MLDTs) are interventions based on the theory of increasing lymphatic circulation thereby improving the removal of biochemical wastes and enhancing fluid dynamics [2]. The term lymphatic pump was invented by Earl Miller, D.O. to describe what was formerly known in osteopathic medicine as the thoracic pump technique [8]. Osteopathic manipulative therapy has several MLDTs dedicated to enhancing lymphatic flow and circulation through the lymphatic system [8, 9]. The pumping vertical rhythmic motion in the supine position increases blood vessel dilatation, increases blood flow, thus increases the metabolism of by-products and waste. MLDTs have been used in a variety of diseased populations that have difficulty with waste removal, such as chronic obstructive pulmonary disease, pneumonia or to assist with wound care [9, 10]. It has not been examined whether MLDTs can help improve recovery after intense exercise faster than previously recognized methods. If effective, this technique may be used as a novel recovery method following intense exercise or therapy.

The primary aim of this study was to examine the pedal pump technique and its effect on acute blood lactate concentrations following maximal exercise compared with passive recovery. Secondarily, this study will examine the effect of the pedal pump technique on acute heart rate (HR), blood pressure (BP) and oxygen consumption (VO_2_) recovery following maximal exercise compared with passive recovery.

We hypothesize that the pedal pump procedure will enhance lymphatic flow during recovery and will produce a more efficient clearance of blood lactate as compared to passive recovery.

## Methods

### Study Design and Participants

This study was approved by the New York Institute of Technology (NYIT) Internal Review Board. Subjects were recruited via flyers posted on campus at NYIT. All participants read and signed an informed consent prior to participation in the study. All testing was conducted at the NYIT College of Osteopathic Medicine (Old Westbury, NY) Center for Sports Medicine. This study was performed in accordance with the standards of ethics outlined in the Declaration of Helsinki.

**Fig. 1:**
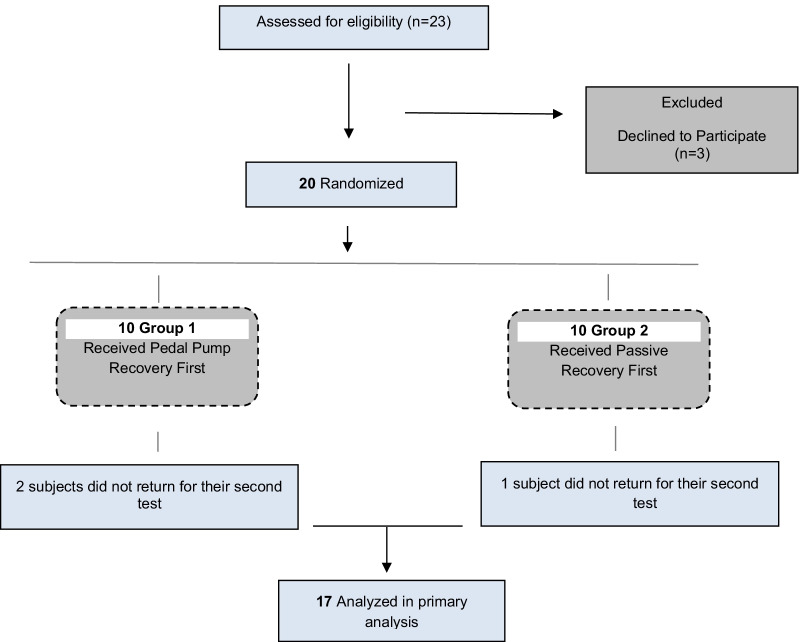
Flow of participants through study

This was a randomized cross-over design experiment that enrolled 20 physically active college students who exercised at least 4 times a week. Three participants did not complete the second trial and were not used in data analysis. Seven women (age 24 ± 0.95) with a body mass index (BMI) of 23 ± 3.01 and 10 male**s** (age 24 ± 1.8) with a BMI of 24 ± 1.8 completed the study (Fig. 1). All participants were in good self-reported health and were administered The Physical Activity Readiness Questionnaire (Par-Q) [11] screen test to assess exercise risk factors. If someone answered yes to any question they were excluded from the study. A power analysis was performed to determine the required sample size. To yield a minimum 0.80 power with an alpha level set at 0.05 would require enrolling at least 14 participants [5].

### Exercise Testing

Participants performed a Bruce Protocol graded treadmill exercise test on two separate occasions. This test consists of a maximum of five 3-min stages on a treadmill [12, 13]. Participants rested supine for 15 min prior to treadmill testing. Resting physiological measures included heart rate (HR), Blood pressure (BP), Oxygen consumption (VO_2_) and blood lactate (BL). The criteria set for peak exercise was any of the following: (1) age-adjusted maximal heart rate attained; (2) a plateau of oxygen uptake was attained; (3) if the subject was unable to maintain the pace of the treadmill; (4) a Respiratory Exchange Ratio (RER) of over 1.3 and/or a plateau in ventilation (Ve) [11]. Additionally, the American College of Sports Medicine (ACSM) guidelines for terminating exercise testing were followed [11]. Participants were verbally encouraged to continue during exercise. All physiological measures were recorded at rest, within 1 min post- peak exercise, and at recovery minutes 4, 7, 10, and 20 post exercise.

### Physiological Parameters

Testing was performed on a COSMED® CPET metabolic cart (COSMED USA Inc. Concord, CA). Heart rate was recorded using a heart rate strap that synced with the COSMED unit. Blood pressure (BP) was measured manually by an experienced clinician (exercise physiologist or physician) using an upright mercury sphygmomanometer unit (W.A. Baumanometer Co. Inc. Copiague, NY, USA). Capillary blood lactate samples were analyzed by the Accutrend® Plus Ltd portable lactate analyzer system (F. Hoffmann-La Roche, Switzerland). Outcome measures of VO_2_, VCO_2_, RER, and Ve were recorded at 1-min intervals throughout testing and recovery as measured by the COSMED unit. Outcome variables that were measured at rest, peak exercise, and recovery minutes 4, 7, 10 and 20 min included HR, VO_2_, BP, and BL.

All participants were randomly assigned using an online program (Research Randomizer www.randomizer.org) to perform either supine passive recovery on the first visit or supine lymphatic pump on the first visit. A minimum of 5 days was required between exercise testing days with a maximum of 10 days between testing days.

### Passive Recovery

When the subject attained peak exercise according the protocol (or to the same parameters as the previous test), the treadmill was brought down from the incline and speed was reduced and all outcome measures were taken within 1 min post peak exercise. The subject was the placed in a supine position for 10 min still wearing the metabolic mask. Outcome measures were taken at minute 4, minute 7, and at the 10-min marker post peak exercise. Following 10 min of lying supine, the subject sat upright in a chair to continue to recover for another 10 min. At minute 20 following peak exercise all outcome measures were taken.

### Pedal Pump Recovery

**Fig. 2:**
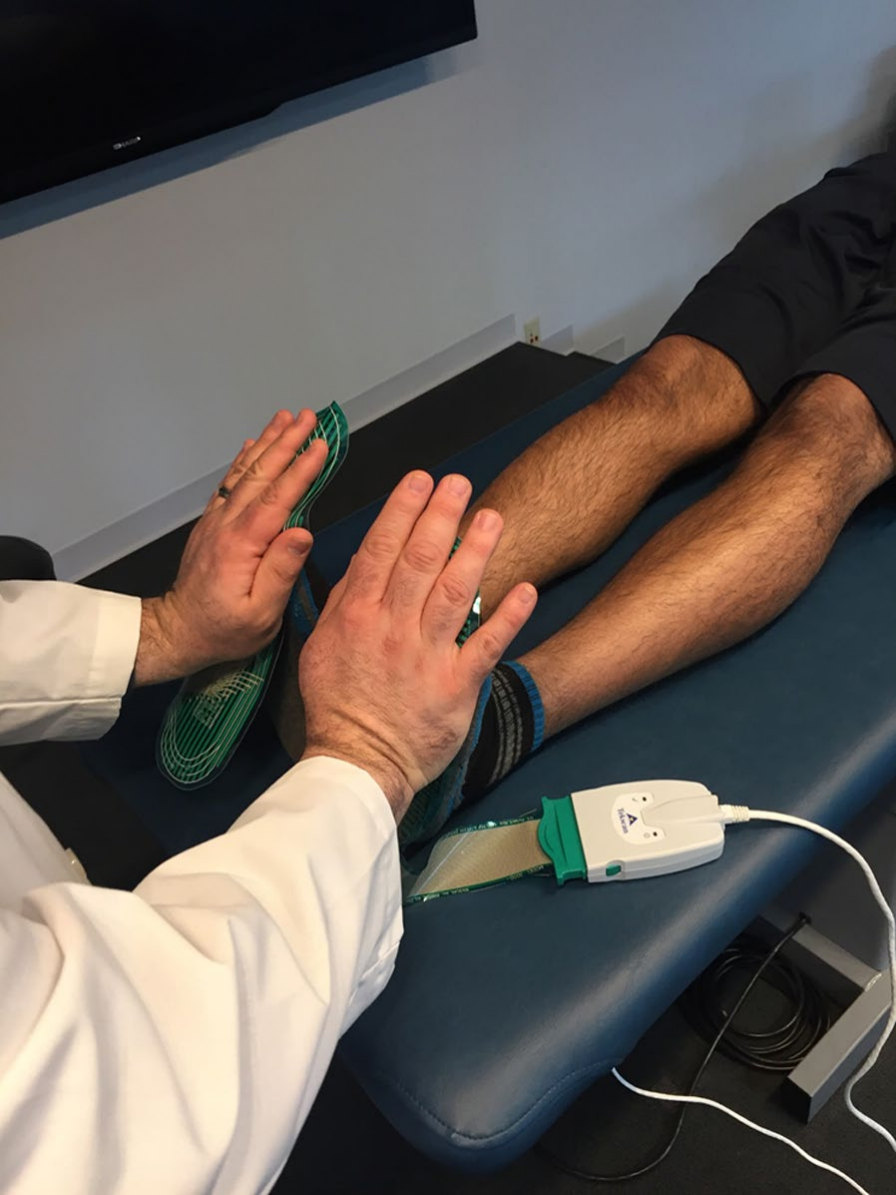
F-scan system to measure force

When the subject attained peak exercise according the protocol (or to the same parameters as the previous test), the treadmill was brought down from the incline and speed was reduced and all outcome measures were taken within 1 min of peak exercise. The subject was the placed in a supine position and the pedal pump technique was performed for 10 min while still wearing the metabolic mask. To standardize the intervention, the pedal pump was performed using Tekscan F-scan system (Tekscan, Inc.® South Boston, MA, USA) to measure the amount of force being applied. The average force applied was 12.8 ± 1.7 lbs. and a metronome was set to 120 cycles/minute to match parameters done in a pilot study using a whole body periodic acceleration [14, 15]. The Tekscan pads were placed on the bottom of the participants feet while the physicians applied the pumping technique (Fig. 2). Outcome measures were taken at minute 4, minute 7, and at the 10-min mark post peak exercise. Following 10 min of supine pedal pump, the subject was sat upright in a chair to continue recovery for another 10 min. At minute 20 following peak exercise all outcome measures were taken.

### Data Analysis

A repeated measures ANOVA was used over time to compare all outcome measures (HR, BP, VO_2_, BL). Significant results from the ANOVA were further examined with a Bonferroni’s post hoc test. Statistical significance was set at *p* < 0.05. All data is presented as mean and standard deviation (SD).

## Results

The mean force applied to each subject during the pedal pump recovery arm was 12.8 ± 1.7 lbs. with an approximate rate of 120 propulsions per minute.

### Primary Outcome

Our primary outcome was BL. There was no difference within subjects at baseline for BL between conditions (2.0 ± 1.1 mmol vs 2.3 ± 1.7 mmol; *p* = 0.6). No changes were found in BL levels during recovery between pedal pump condition and resting condition at minute 1 (9.5 ± 3.8 mmol vs 10.5 ± 2.5 mmol; *p* = 0.5), minute 4, (9.8 ± 4.5 mmol vs 9.8 ± 3.2 mmol; *p* = 0.6), minute 7, (2.0 ± 1.1 mmol vs 2.3 ± 1.7 mmol; *p* = 0.07), or minute 10 (2.0 ± 1.1 mmol vs 2.3 ± 1.7 mmol; *p* = 0.5). However, there was significant differences in BL at minute 20 between conditions (pedal pump (4.7 ± 1.2 mmol, passive (6.3 ± 1.7 mmol; *p* = 0.04).

### Secondary Outcomes

**Fig. 3:**
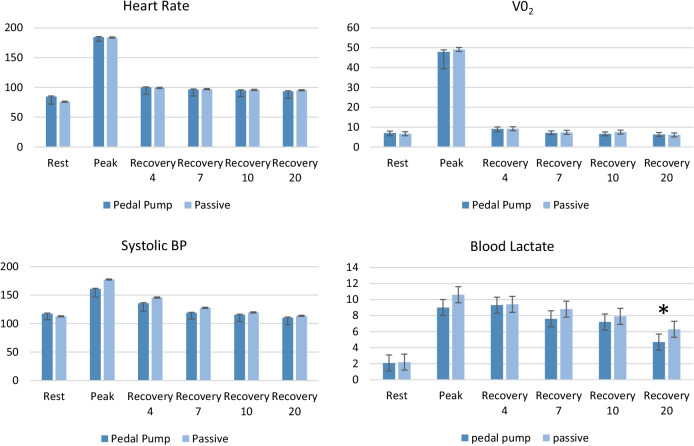
Physiological outcome measures between pedal pump and passive recovery at rest, peak exercise, and recovery minutes 4, 7, 10 and 20. *Significance (*p* = 0.04)

All subjects attained the same duration of exercise on both testing days and there were no significant differences in any outcome measures within subject at baseline or at peak exercise HR, SBP, VO_2_ or BL) when comparing the two days, these values are presented in Fig. 3.

There were no changes found in any secondary outcome measures at rest, peak exercise, and at minutes 1, 4, 7, 10 (HR, SBP, VO_2_, *p* > 0.05).

## Discussion

The aim of this study was to compare the pedal pump technique to passive recovery following maximal exercise testing. The results showed that at minute 20 following peak exercise, the pedal pump resulted in significantly lower blood lactate levels with no changes in exercise markers in recovery in HR, SBP, and VO_2_ between the two methods.

Blood lactate build up is the leading cause of fatigue during exercise. The increase in BL accumulation increases H^−^ production. This in turn reduces action potential which decreases calcium secretion from the sarcoplasma and muscle activity [16]. Peak lactate concentrations may occur at various times following post exercise. This is influenced by the intensity of the exercise, the muscle’s involved and the duration of the activity [5, 17, 18]. Blood lactate recovery in trained individuals declines linearly following peak concentrations. Typically peak blood lactate concentrations is seen between minute 1 and minute 5 of recovery, at which time lactate clearance may begin [17]. In order to determine the duration it takes to return to baseline BL concentrations is dependent upon the level of fitness of each individual, genetics, and recovery method. The net rate of blood lactate removal is dependent upon the recovery method. Based on previous literature, the average clearance rate of plasma blood lactate during inactive recovery following peak exercise is approximately .30 mmol/L·min [19]. Although it has been shown that active recovery is more effective in blood lactate removal than passive recovery, passive recovery has shown to improve HR and Excess Post Oxygen Consumption (EPOC) more rapid than active recovery following high intensity exercise [5, 20]. Although a more rapid HR and EPOC recovery is ideal for a recovering athlete, dissipating blood lactate during early recovery is important to prevent delayed DOMS [3]. In certain circumstances, DOMS can be described as a mild form of rhabdomyolysis. Rhabdomyolysis refers to the disintegration of striated muscle which results in the release of muscular cell constituents into the extracellular fluid and circulation. Exertional rhabdomyolysis is occasionally seen after strenuous exercise.

Passive recovery in a supine position has nearly zero venous pressure due to the decreased force of gravity. However, when in a standing or sitting position hydrostatic pressure is increased as a result of gravity Throughout active recovery there is an increase in blood flow and lymphatic pumping due the veno-muscular pumping mechanism, which allows for lower venous pressure following each rhythmic muscular contraction that pushes blood in the direction of the heart against gravity when upright [6, 7]. By increasing blood flow to the muscles and systemic blood flow, lactic acid metabolism is increased via oxidation and gluconeogenesis [21].

Other methods to reduce blood lactate accumulation and DOMS that are frequent in athletes are compression garments, massage, or cryotherapy [1, 22, 23]. There is some evidence that cold water immersion or cryotherapy can reduce DOMS when compared to passive methods, however, there is insufficient evidence comparing it to other methods [24, 25]. These methods can also be impractical, costly, and unpleasant for the participant. Cold water submersion typically involves the subject being submerged to waist level, but some research has explored submersion as high as the sternum or shoulders. This method commonly uses temperatures between 10 and 15 °C with a submersion time that can range from 5 to 25 min in intermittent bouts of submersion. Most research has explored treatment duration approximately for 12 min [25].

There were limitations to this study that included failure to follow up with our subjects to objectively collect data to observe fatigue or soreness 24 or 48 h post testing as well as collecting subject feedback after each method of recovery.

This study has several implications. First, it investigated the average force that is applied by multiple investigators who are trained in performing the lymphatic pump technique. To date there are no qualitative data suggested to perform this technique. Franzini et al. suggests a low velocity moderate amplitude of force, at approximately a rate of 120 propulsions per minute for two minutes [15]. A metronome was set to 120 cycles/minute to match Franzini’s suggestions and the parameters done in a pilot study using a whole body periodic acceleration [14, 15]. To our knowledge this is the first time force while performing the pedal pump was captured quantitatively to offer an effective replication of the methods. Secondly, this is the first time this specific lymphatic pumping technique was observed in an exercise recovery capacity. Future investigation into lymphatic pumping techniques may offer a more practical treatment option than other alternative methods currently being used.

## Conclusions

The results from this project demonstrate that the pedal pump technique at a standardized force and frequency significantly reduced blood lactate accumulation 20 min post maximal exercise testing as compared to passive recovery. The effectiveness of MLDTs to improve lymphatic function, especially in the area of sports medicine warrants further investigation.

## Data Availability

Data available upon request.
